# Effects of epidural analgesia exposure during parturition on autism spectrum disorder in newborns: A systematic review and meta-analysis based on cohort study

**DOI:** 10.3389/fpsyt.2022.974596

**Published:** 2022-09-06

**Authors:** Xiaobo Wang, Jie Li, Dezhao Liu

**Affiliations:** The Fifth Affiliated Hospital, Sun Yat-sen University, Zhuhai, China

**Keywords:** labor epidural analgesia, autism spectrum disorder, meta analysis, systematic review, epidural analgesia

## Abstract

**Background:**

Labor epidural analgesia (LEA) is one of the most effective and widely used approaches for pain relief during parturition. we conducted this meta-analysis to evaluate the association between LEA exposure during parturition and ASD in newborns.

**Methods:**

PubMed, Cochrane Library, EMBASE and Web of Science database were searched from inception to January 2, 2022 to identify related articles. Literature screening was carried out strictly according to the inclusion and exclusion criteria, and data were extracted and imported into STATA 15.0 software for meta-analysis.

**Results:**

A total of 5 studies with 1763454 participants were included. A statistically significant correlation was observed between LEA and changed ASD (RR = 1.20, 95%CI (1.17, 1.24)), and the correlation between LEA and ASD were analyzed by univariate HR (ES = 1.34, 95%CI(1.27,1.40), *P* < 0.05) and Multivariate HR (ES = 1.13, 95%CI (1.05,1.21), *P* < 0.05).

**Limitation:**

First, few studies were included, and most patients were from the United States. Second, the included studies were observational cohort designs, which cannot avoid selection and measurement bias. Third, the results of the included studies were heterogeneous, and a more detailed subgroup analysis was not possible.

**Conclusion:**

There is a correlation between LEA during parturition and the risk of ASD in newborns. The Newborn whose mother received LEA during her birth-giving might be more likely to develop ASD.

## Introduction

Parturition pain refers to severe pain caused by intense uterine contractions and the subsequent peri-uterine tissue damage during parturition, which is one of the important causes of parturition phobia for an expectant mother (1, 2). The intense pain during parturition leads to increased secretion of catecholamines and other substances, resulting in complications such as fetal distress. It could also lead to cesarean section, which has adverse effects on postpartum recovery, and the surgery scar on the corpus uteri would be a significant risk for second pregnancy (3, 4). Therefore, alleviating maternal pain during parturition has become an urgent problem to be solved (5). Currently the labor epidural analgesia (LEA) is highly valued by clinicians, which refers to the injection of anesthetics into epidural cavity to temporarily block the nerve conduction of in spinal ganglion, so as to avoid the complications due to overpowering pain during parturition (6). LEA is one of the most effective and widely used anesthetic approaches for pain relief during labor (7). In United States, 70% of the expectant mothers receive LEA during their birth-giving (8), and it is demonstrated to be safe for the newborns (9). Our previous study explored a possible association between LEA and long-term adverse neurodevelopment in newborns, and the results indicated autism spectrum disorder (ASD) (10, 11). ASD is a term used to describe a range of early-onset social communication deficits, and is also used on the repetitive sensory–motor behaviors related to genetics (12). From 2002 to 2016, the incidence of ASD in the United States increased from 0.66 to 1.85%, leading to the emerging of studies exploring the relationship between ASD and genetics, maternal and nervous system (13), and these studies has reached some depth for finding several risk factors that might contributed to ASD and neurological diseases in newborns: birth trauma, low birth weight, and caesarean section (14). The relationship between the risk of ASD and intrapartum interventions, including labor epidural analgesia (LEA), has been the current research hotspot. We previously conducted a meta-analysis, including 61 studies and 20 million parturitions, and the results indicated that ASD is strongly associated with caesarean section (15). However, the risks associated with neuraxial anesthesia commonly used for routine vaginal delivery have not been addressed (16, 17), and several recent studies have come to the opposite conclusion for the association between ASD and LEA (10, 18–20). As LEA is the current the standard strategy for vaginal delivery pain management, it is important to assess the effect of maternal LEA on the newborns. Therefore, we conducted this meta-analysis using prenatal and delivery data related to ASD diagnosis, so as to evaluate association of neonatal ASD with the exposure to LEA.

## Materials and methods

This meta-analysis was registered on the International Prospective Registry of Systematic Reviews (Registration No. CRD42022303695) and was reported according to the Preferred Reporting Item for Systematic Reviews and Meta-Analyses (PRISMA) statement.

### Selection criteria and search strategy

PubMed, Cochrane, EMBASE, and Web of Science databases was systematically searched, from inception to January 2, 2022, for published studies, with no language restrictions. We used the following combined text and MeSH terms: “Analgesia, Epidural” and “Autism Spectrum Disorder “. The complete search used for PubMed was: ((”Analgesia, Epidural”(Mesh)) OR ((Analgesia, Epidural(Title/Abstract)) OR (Epidural Analgesia (Title/Abstract)))) AND ((”Autism Spectrum Disorder”(Mesh)) OR ((((Autism Spectrum Disorder (Title/Abstract)) OR (Autism Spectrum Disorders (Title/Abstract))) OR (Autistic Spectrum Disorder (Title/Abstract))) OR (Disorder, Autistic Spectrum (Title/Abstract)))). The reference lists of retrieved articles were also searched for potential eligible studies.

### Inclusion and exclusion criteria

Studies that meet the following criteria were included:

(1) Types of study: original studies without restriction in study design;
(2) Types of participants: singleton children born naturally;
(3) Types of exposure: exposure to LEA during the birth-giving.

Pediatric-only studies, conference abstracts, case reports, editorial letters, and other unpublished studies were excluded.

(4) Outcome and measures: The outcome was clinical diagnosis of ASD. ASD is assessed via Autism Diagnostic Interview, Revised (ADI-R), Autism Diagnostic Observation Sheet (ADOS), Autism Diagnostic Observation Sheet, or the second edition of ADOS-2. The outcome measure was the presence or absence of ASD in the LEA group and in the non-LEA group. Moreover, cox proportional hazards model was used to analyze the relationship between maternal LEA and ASD.

Exclusion criteria were the following:

(1) Studies lacking key information such as hazard ratio (HR), 95%CI and P value;
(2) Non-English language;
(3) *In vitro* and animal studies;
(4) Reviews, commentaries, editorials, protocols, case reports, qualitative research, or letters.

### Data extraction and quality assessment

Data extraction and quality assessment were conducted by two reviewers (Xiaobo Wang, Jie Li) independently, and any disagreements were settled by a third reviewer. We extracted the following data from each selected study: first author name, publication year, country, sample size, baseline characteristics of participants and other relevant data. The endpoints were also extracted: the number of children diagnosed with ASD during the follow-up period in the LEA group and in the non-LEA group; crude risks of ASD(HR), or adjusted risks of ASD(HR) and 95% confidence intervals (95%CI). Qualified case-control studies were assessed according to Newcastle-Ottawa Scale (NOS) (21), and the NOS contains eight items, which are categorized into the three dimensions of selection, comparability, and exposure (case control studies). The full score was 9 points, and studies with scores ≥6 points were considered to be of high quality (22). Any discrepancies were resolved by consensus and arbitration by a panel of investigators within the review team.

### Statistical analysis

To find the relationship between ASD and LEA, Stata 16.0 software was used for statistical analysis. This meta-analysis calculated the pooled OR or HR with its corresponding 95% CI to assess the relationship between maternal LEA and ASD. The heterogeneity of the included studies was tested by Q test and I2. If I2 < 50% it suggested that there was no heterogeneity among the studies, and fixed effect model was used for analysis. On the contrary, if I2 > 50%, it suggested that the studies were heterogeneous and random effect model was used for analysis. Sensitivity analysis was used by removing individual studies to improve the robustness of the pooled results. Moreover, we also used meta-regression analysis when exploring heterogeneity between studies. *P* values in the meta-regression revealed the overall significance of the factors’ influence. Additionally, *P* values from the meta-regression were inversely proportional to the degree of heterogeneity; *P* values less than 0.05 indicate factors that could be an important source of heterogeneity. The likelihood of publication bias was assessed by visual inspection of funnel plot for study size against treatment effect. Begg’s and Egger’s tests were used to detect publication bias.

## Results

### Literature retrieval process and results

**FIGURE 1:**
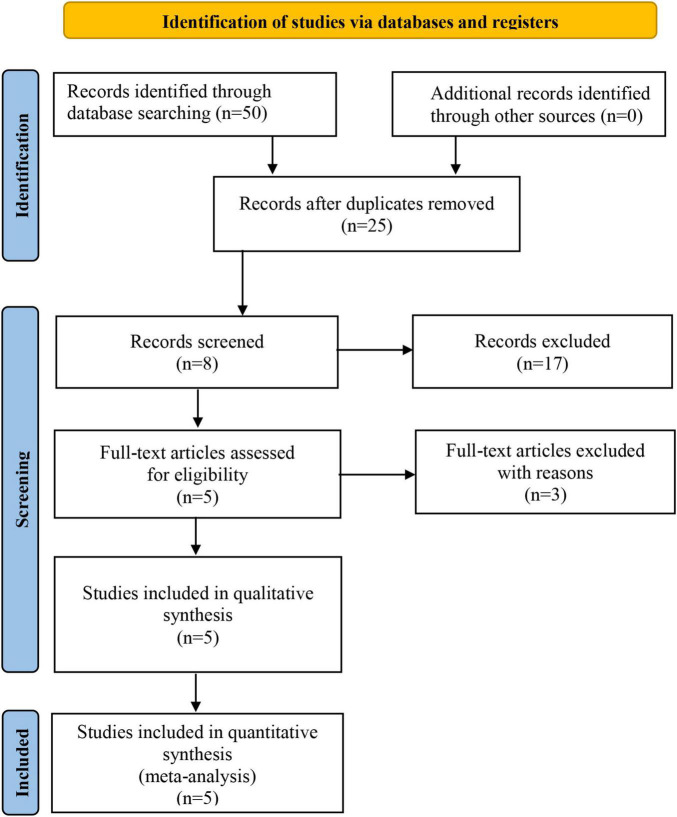
Flow chart of literature screening.

There were 50 articles identified through initial search. Based on titles and abstracts, 17 articles were excluded due to irrelevant study-design, and 3 articles were excluded because of repeated publication, full text unavailable, data unavailable. Finally, a total of 5 articles with 1763454 participants were included. Searching process is shown in Figure 1.

### Characteristics of included studies

All the 5 included studies (10, 17–20) were retrospective cohort-design with a total of 1763454 participants were published in English. All the included studies were of high quliaty with NOS scores of 7. Detailed characteristics of included studies are shown in Table 1.

**TABLE 1:** Basic characteristics of the included literature.

References	Sample size	Age of women, mean (SD/range)			Offspring risk of ASD, HR (95% CI)	
		Study design	Exposed	Unexposed	Crude	Adjusted
Gillian et al. (17)	388 254	Retrospective Cohort study	29.9 (5.5)	30.1 (5.5)	1.32 (1.24–1.40)	1.09 (1.00–1.15)
Anders et al. (10)	479 178	Retrospective Cohort study	30.0 (26.6–33.5)	31.1 (27.8–34.4)	1.29 (1.21–1.37)	1.05 (0.98–1.11)
Chun et al. (20)	147 895	Retrospective Cohort study	29.8 (5.6)	30.6 (5.5)	1.48 (1.34–1.46)	1.37 (1.23–1.53)
Tai et al. (19)	624 952	Retrospective Cohort study	29 (13–52)	30 (13–54)	1.38 (1.31–1.46)	1.11 (1.04–1.18)
Elizabeth et al. (18)	123 175	Retrospective Cohort study	28.2 [5.8]y		1.25 (1.15–1.36)	1.08 (0.97–1.20)

### Quality assessment

All the included studies were of high quality with NOS scores of 7. Each study did not score on the fourth item (Demonstration that outcome of interest was not present at start of study) on “selection”. All studies controlled for important confounders. All included studies had scores greater than 6 and were considered to be of high quality. The detailed scoring results are shown in Table 2.

**TABLE 2:** NOC scores of included literature.

References	Representativeness of the exposed cohort	Selection of the non-exposed cohort	Ascertainment of exposure	Demonstration that outcome of interest was not present at start of study	Comparability of cohorts on the basis of the design or analysis	Assessment of outcome	Was follow-up long enough for outcomes to occur	Adequacy of follow up of cohort	Total scores
Gillian et al. (17)	1	1	1	0	1	1	1	1	7
Anders et al. (10)	1	1	1	0	1	1	1	1	7
Chun et al. (20)	1	1	1	0	1	1	1	1	7
Tai et al. (19)	1	1	1	0	1	1	1	1	7
Elizabeth et al. (18)	1	1	1	0	1	1	1	1	7

### Result of meta-analysis

**FIGURE 2:**
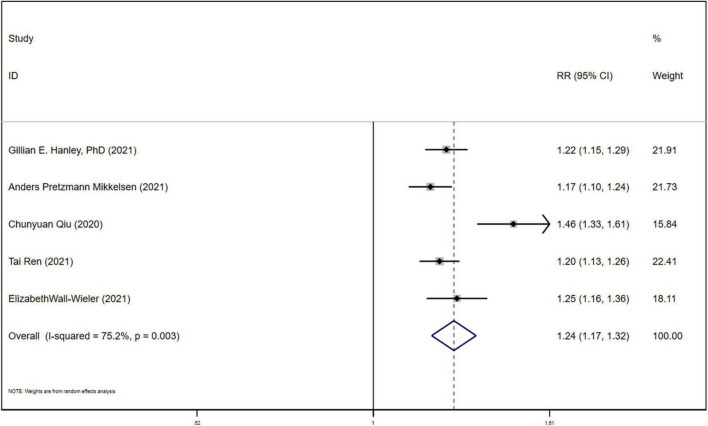
Forest plot for the association between LEA and ASD.

**FIGURE 3:**
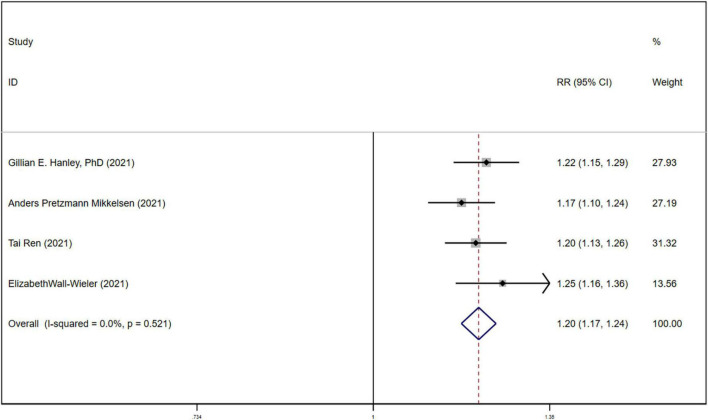
Forest plot for association between LEA and ASD after removing Qiu.

All the 5 studies (10, 17–20) reported the association between LEA exposure during parturition and ASD, using heterogeneity tests (*P* = 0.003). Meta-analysis showed that the incidence of ASD in the newborns in LEA-exposed group was significantly higher than those in non-exposed group (RR = 1.244, 95%CI (1.171-1.322), *P* < 0.05), as shown in Figure 2. Sensitivity analysis found that after removal of the study by QIU (20), the heterogeneity was eliminated (*P* = 0.521; I2 = 0.0%), suggesting that the heterogeneity was from Qiu (20). We found that compared to other studies, the non-LEA group sample size of QIU (20) is significantly less than others. After eliminating this study, the fixed effects model was used to perform a second meta-analysis on the outcome, and the results still showed that the incidence of neonatal ASD in the LEA group was significantly higher than that in the non-LEA group RR = 1.203, 95%CI (1.167, 1.240), *P* < 0.05, as shown in Figure 3. The results did not reverse suggesting the stability and reliability.

**FIGURE 4:**
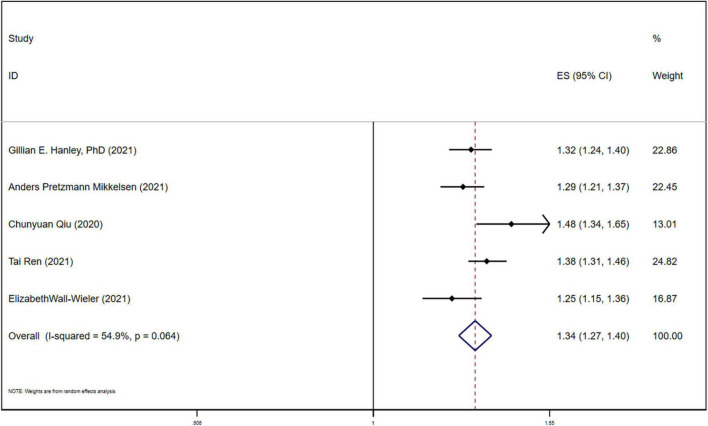
Forest plot for univariate HR of LEA and ASD.

The association between LEA and ASD was analyzed by univariate HR analysis and heterogeneity test was performed (*P* = 0.064; I2 = 54.9%), the random effect model was used for analysis, and the results showed that the combined effect size (ES 1.34, 95%CI (1.27, 1.40), *P* < 0.05) were shown in Figure 4. The results of meta-analysis showed that there was statistically significant heterogeneity among the studies, so meta-regression analysis was performed, and this showed that the source of heterogeneity arose from the sample size of the LEA group and the non-LEA group across the included studies, *P* < 0.05.

**FIGURE 5:**
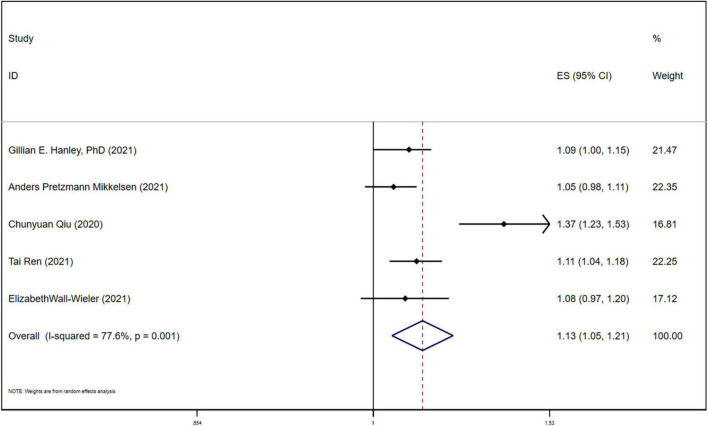
Forest plot for multivariate HR of LEA and ASD.

After adjusting for potential confounders, including birth year, medical center, maternal age at delivery, parity, race/ethnicity, educational level, household income, history of comorbidity, diabetes during pregnancy, smoking during pregnancy, preeclampsia or eclampsia, prepregnancy body mass index, gestational weight gain, gestational age at delivery, and birth weight. Multivariate HR analysis of the association between LEA and ASD showed heterogeneity (*P* = 0.001; I2 = 77.6%), the random effect model was used for analysis, and the results still showed that maternal LEA was associated with increased ASD risk in children (ES 1.13, 95%CI (1.05, 1.21), *P* < 0.05) Figure 5. Meta regression was used to test the sample size in the LEA group and non-LEA group, the average age of pregnant women and fetal sex across the included studies, and the p value was obtained. The sample size in the exposure group and non-LEA group may be the source of heterogeneity, while the average age of pregnant women and fetal sex(male) are not the source of heterogeneity.

### Sensitivity analysis

**FIGURE 6:**
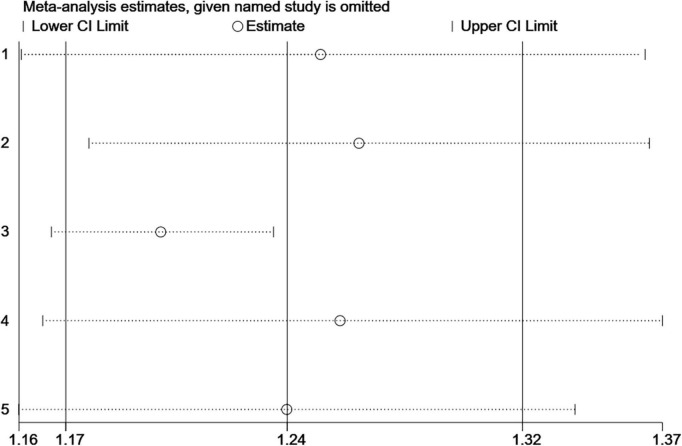
Sensitivity analysis for the association between LEA and ASD.

This study evaluated the results of meta-analysis on LEA exposure through sensitivity analysis and found that the circles representing each study were within two edges, indicating that the conclusions of this meta-analysis were stable and reliable (Figure 6).

### Publication bias

According to the Cochrane guidance, publication bias should not be used since there is only 5 articles included. Therefore, we no longer conduct the analysis of the funnel plot in this study.

## Discussion

Labor analgesia technology is the measure of economic development and spiritual civilization of a country, in which the promotion level of labor analgesia technology is a yardstick. It also means the improvement of women’s social status, humanistic care in medical care and the continuous improvement of the medical system (23). It is indisputable that the adverse effects caused by parturition pain on maternal physiology and psychology, which can lead to maternal neuroendocrine response, decreased placental blood flow, risk of fetal distress and neonatal asphyxia (24).

In this study, a meta-analysis was conducted to demonstrate that the newborns exposed to LEA during parturition were 1.24 times more likely to develop ASD than those unexposed, which may be related to the pathogenesis and progression degree of ASD and the use of anesthetic drugs. While Glezerson (25) found that maternal exposure to LEA was associated with a 37% increased risk of ASD in children. Longer epidural exposure was associated with a greater risk of ASD, with a 33% increase of risk in less than 4 h of LEA exposure, a 35% increase in 4 to 8 h of LEA exposure, and a 46% in over 8 hours exposure, compared with the unexposed group. Qiu et al. (26) also found that the risk of ASD in the newborns increased with increasing doses of anesthetics, which may be associated with neurodevelopmental abnormalities in newborns caused by short exposure to LEA (27). Through multivariate analysis indicated that the occurrence of ASD in LEA was also closely related to family income, fetal number, fetal size, number of births, and maternal blood pressure.

Autism is a heterogeneous neurodevelopmental condition characterized by early-onset social dysfunction and abnormally restricted, repetitive behaviors and interests (28), with a global prevalence of approximately 1%. Autism is more common in males, and 70% of the patients have multiple comorbidities (29). Patients with autism have atypical cognitive features such as impaired social cognition and social perception, executive dysfunction, and atypical perception and information processing (30). These features are manifestations of atypical nervous system development at the system level. Genetics plays a key role in the pathogenesis of autism, combined with environmental factors in early development. Both minor mutations with large effects and common mutations with small effects would increase the risk (31). Epidural analgesia is wildly used during parturition, whereas most anesthetics are neurotoxic, mainly by inducing neuronal cell death, which can lead to behavioral and cognitive abnormalities (32). In juvenile animal models, anesthesia exposure may affect synapse formation, neuronal apoptosis, and glial cell development (33).

There is currently little evidence for the association between epidural analgesia during labor and autism, and previous research has not been able to reliably confirm or refute this potential association. Qiu et al. (20) evaluated cohort studies involving 14,795 only children and found that epidural analgesia during parturition was associated with autism. The adjusted OR was 1.37 (95% CI, 1.23-1.53), and the main results of the study were close to the rough estimates of the present study, while some factors such as maternal psychiatric history or use of psychotropic medications and family history of autism were not considered in that study. A Canadian study included 12,175 only children and adjusted for perinatal factors such as increased labor, dystocia, and fetal distress, the results showed no association between epidural analgesia during labor and autism in children (OR, 1.08 (95% CI, 0.97-1.20)) (18).

Although this meta-analysis demonstrated an association between LEA and ASD, it also had some limitations. First, the included studies are few. Despite sufficient sample size, most of the patients were from United States. The lack of diversity of study populations, such as the lack of African and Asian populations, resulted in unsatisfied external validity. Second, the included studies are observational cohort-design, selection and measurement bias could not be avoided. Third, heterogeneity exists among the results of included studies, to which different sample sizes, different control conditions in the control group, different diagnostic criteria and the influence of various confounding factors might contribute, while a more detailed subgroup analysis cannot be performed due to the limited number of studies. Follow-up studies should take into accounts these problems to reach a more robust conclusion.

## Limitations

Although this meta-analysis demonstrated an association between LEA and ASD, it also had some limitations. First, the included studies are few. Despite sufficient sample size, most of the patients were from United States. The lack of diversity of study populations, such as the lack of African and Asian populations, resulted in unsatisfied external validity. Second, the included studies are observational cohort-design, selection and measurement bias could not be avoided. Third, heterogeneity exists among the results of included studies, to which different sample sizes, different control conditions in the control group, different diagnostic criteria and the influence of various confounding factors might contribute, while a more detailed subgroup analysis cannot be performed due to the limited number of studies. Follow-up studies should take into accounts these problems to reach a more robust conclusion.

## Conclusion

In conclusion, there is a certain relationship between LEA and ASD. For pregnant women who have undergone epidural analgesia during clinical parturition, their newborns should be screened as early as possible for ASD, so as to achieve early detection and intervention, and to improve the quality of life of patients and their newborns as much as possible.

## Data availability statement

The original contributions presented in this study are included in the article/supplementary material, further inquiries can be directed to the corresponding author/s.

## Author contributions

DL and XW conceived of the presented idea. XW and JL wrote the main manuscript text and collected and analyzed the data. DL played an important guiding role in revising the manuscript. All authors discussed the results and contributed to the final manuscript.
